# Influenza

**Published:** 1861-04

**Authors:** 


### Influenza.

We have for several years relied, almost entirely, upon opium in the treatment of the various degrees of "bad cold," known as Coryza, acute bronchitis, catarrh, influenza, &c. Even in the more violent epidemic form of influenza, in which fever, and sometimes prostration are attendants, the preparations of opium are mainly relied upon. The use of nourishing and invigorating food and drinks are also to be encouraged. The views of Dr. Christison on this subject, as found condensed in the Epitome of Braithwait's Retrospect, is as follows:

"*Opium.*—Dr. Christison finds opium of the most use in inflammations of the mucous membranes, especially coryza, catarrh, influenza and dysentery. He would cure coryza at once by causing the patient to avoid all meals after dinner, to use liquid sparingly, and by giving a full dose of muriate of morphia or Battley's solution at bed-time; and breakfast before getting up the next morning. It seems necessary, however, that the remedy should be administered early in the attack. 'Febrile catarrh, too, may be checked abruptly in the same way, if patient and physician are lucky enough to meet during the first or second day at farthest.' "

"Dr. Christison has repeatedly seen epidemic influenza thus checked at the outset—the local inflammation vanishing, while the strange lascitude, listlessness and ennui, so characteristic of this disorder, went on as usual for some days during convalescence."

We often see ipecac and other nauseant and relaxing agents set down as the means of relieving these annoying disorders, but, although almost complete relief is immediately obtained from them often, a speedy relapse is almost as certain to follow in a few hours.

